# Berengario da Carpi and the Renaissance of Brain Anatomy

**DOI:** 10.3389/fnana.2019.00011

**Published:** 2019-02-13

**Authors:** André Parent

**Affiliations:** Department of Psychiatry and Neuroscience, Faculty of Medicine, Université Laval, Quebec, QC, Canada

**Keywords:** history of neuroscience, italian renaissance, neuroanatomy, brain surgery, *rete mirabile*, *anatomia sensibilis*, brain anatomy, anatomical illustrations

## Abstract

Berengario da Carpi (Jacopo Barigazzi) was born around 1460 in the small Italian town of Carpi near Modena. Berengario’s father, Faustino, was a reputable barber-surgeon who initiated his son early into the art of anatomy and surgery. After his graduation from the University of Bologna in 1489, Berengario rapidly acquired an enviable reputation as a physician and surgeon following the successful treatment of several dignitaries, including Lorenzo de’ Medici, Duke of Urbino who suffered a severe head injury in 1517. While professor of anatomy and surgery at the University of Bologna, Berengario published in 1518 his *De fractura cranei*, a landmark work on cranio-cerebral surgery. Berengario’s masterpiece, however, is undoubtedly his detailed *Commentaria* on the famous medieval anatomy treatise of Mondino de’ Liuzzi (ca. 1270–1326) that he published in 1521. A shorter version entitled *Isagogae Breves* appeared a year later. Besides a facsimile of Mondino’s work, Berengario’s *Commentaria* contains a wealth of new information, including observations that challenged Galenic physiology. Galen taught that the *rete mirabile*—a vascular plexus believed to occur at the basis of the human brain—is the locus where the vital spirit is transformed into the more sophisticated animal spirit that is stored in the brain ventricles to be later released at the periphery through a journey within hollow nerves. Courageously, Berengario wrote that despite many attempts he was unable to detect the famous *rete mirabile* in humans. He also noted that the nerves linked to the brain are solid structures, not hollow tubes, as advocated by Galen. His conclusions were based on a systematic dissection method that he called *anatomia sensibilis*, a term that emphasizes the sensory over textual versions of the truth. Berengario contributed significantly to human brain anatomy, with a detailed description of the meninges and cranial nerves and the first comprehensive view of the ventricular system, including choroid plexuses, interventricular foramen, infundibulum, pituitary stalk and gland. Berengario, who died around 1530 in Ferrara, should be remembered for his catalyzing role in the transmutation of medieval morphological knowledge into a modern anatomical science based upon direct observation and experimental demonstration.

## A Short Biography

After a millennium-long stasis dominated by the humor theory brought to us by the famous Greek physician and anatomist Galen (Claudius Galenus, ca. 129–216 AD), the study of human anatomy was restored in the northern part of Italy during the late Middle Ages and early Renaissance. Northern Italy was then benefiting from a progressive rehabilitation of human cadaver dissection put under the veil since the Alexandrian period (3rd century BC). This favorable conjuncture allowed Italians scholars to shed a new light on the fabric of the human body, to the point that Renaissance anatomy is often considered an Italian science (Lind, 1959).

**Figure 1:**
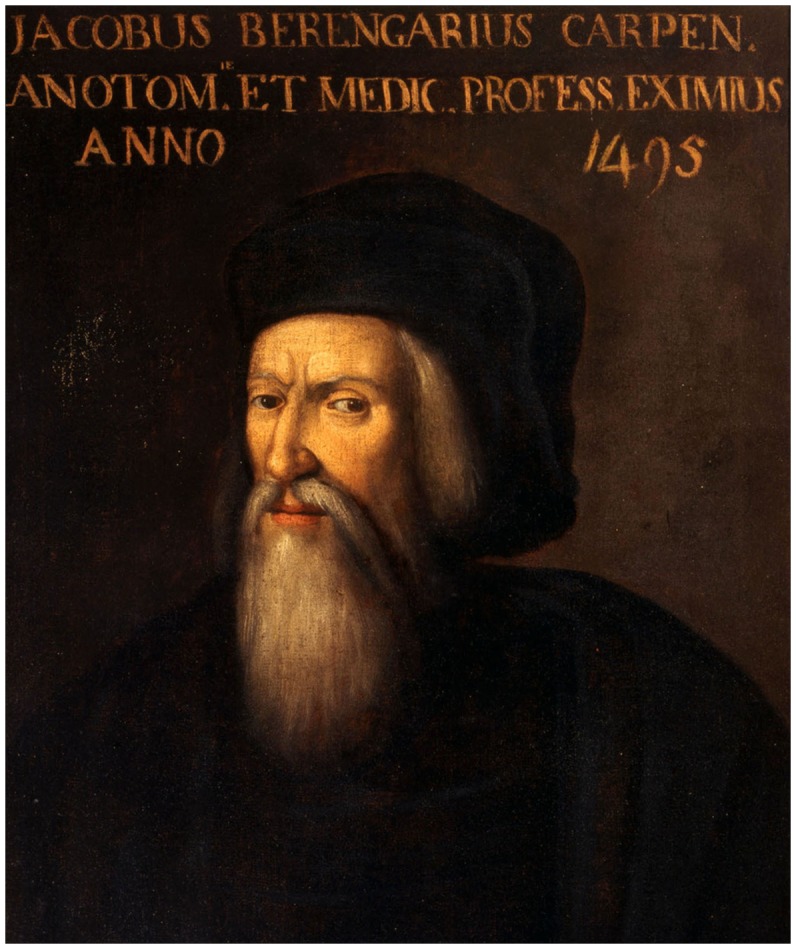
A portrait of Berengario da Carpi from a 16th-century-painter known only by his initials (F. E.). The indication “*Anno 1495*” above the portrait sheds doubt on the identity of the personage illustrated here. At the end of the 15th century, Berengario would have been around 35–40 years of age, whereas the painting shows a rather old man wearing a long beard. Nevertheless, the highly determined and yet ambiguous look of the personage could have been part of Berengario’s physiognomy. The painting is currently displayed in the Museum of the beautiful Palazzo dei Pio, at Carpi, Province of Modena (Courtesy of the *Museo municipale*, Carpi, Italy).

Jacopo Berengario da Carpi (Jacopo Barigazzi), often called simply Carpi or Carpus, was one of the most important anatomists of that period. Berengario (Figure 1) was born around 1460 in the small town of Carpi in the Emilia Romagna region of northern Italy at about 15 km from Modena. His father, Faustino Barrigazi, was a reputable barber-surgeon who initiated his son early to the art of anatomy and surgery. Berengario spent his youth at Carpi with his family at the service of the Pio family headed by Lionello I Pio di Savoia (ca. 1440–1480), Lord of Carpi. There is evidence that in the 1470s the famous humanist and printer Aldus Manutius (ca. 1449–1515) taught to both Berengario and the son of Lionello, Alberto (1475–1531), who later succeeded his father as Lord of Carpi under the name Alberto III Pio di Savoia (Putti, 1937; Lind, 1990; Merlini et al., 2003). The medical knowledge acquired by Berengario at Carpi appears to derive exclusively from experience, but the presence of Manutius is likely to have been instrumental in the opening of his mind to humanities and foreign languages, particularly Latin that he used extensively in his writing.

A major political and financial dispute occurred among several members of the Pio family in the 1480s. Ercole I (1431–1505), Duke of Ferrara, who belonged to the illustrious House of Este, was called in as a peacemaker, but instead he exploited the situation and finally proclaimed himself the new leader of Carpi. Despite the change of political regime at Carpi, Berengario remained loyal to the Pio family. He even spoke openly and insultingly against the Duke of Ferrara, so that he was condemned to pay a fine of 100 ducats or have his nose cut off for his bold words. Berengario’s nose remained untouched because his father accepted to pay the fine, but the event led Berengario to realize that he was no longer welcome in his native town. As a man living in a particularly turbulent period, Berengario did not hesitate to use knowledge as an effective weapon to acquire power or money. There are several episodes in his life that suggest a fierce and ruthless character. For example, while in Bologna, he took part in violent quarrels and assaults and was convicted of robbery, but thanks to some of his protectors he always escaped punishment. As Vittorio Putti (1880–1940), the great authority on the life and work of Berengario da Carpi, put it: “avarice and cupidity arouse in the spirit of this great man stronger passions than glory or virtue” (Putti, 1937).

At the very beginning of the 16th century Berengario moved to Bologna, a city that he called *mea altrice*, the nurse of my learning, and where he had received his medicine doctorate in 1489. His remarkable skill at surgery together with a great capacity to foster several powerful friends worth him the nomination of Lecturer in Surgery in 1502, a post difficult for a foreigner in Bologna to attain. In 1504, Pope Julius II (Giuliano della Rovere, 1443–1513) granted him Bolognese citizenship, hence confirming his professorship at the University of Bologna where he taught during nearly a quarter of a century. All contemporary accounts indicate that Berengario was an excellent and highly popular teacher (French, 1985). Soon after he received his Bolognese citizenship, Berengario got married and shortly thereafter a daughter, Faustina, was born to him, but she died before he wrote his last will (1528). His father passed away in 1517 and Berengario inherited the property of his paternal aunt (Putti, 1937).

Because of his well-earned fame and standing as well as his close relationship with the Papal Court, Berengario was often permitted to leave Bologna to attend some illustrious patients living in other cities, including Florence and Rome. For example, after the onset of the so-called *French Disease* in 1494, he traveled several times to Rome, where he was one of the first to use guaiac and mercury fumigations and ointments to treat distinguished patients suffering from syphilis. Despite its limited clinical success, this procedure brought him fame and richness.

Berengario published his three major medical treatises in a very short period of time, that is, between 1518 and 1522 (see below), while remaining very active and highly successful in the fields of practical medicine and surgery. In the early years of the reign of Pope Clement VII (Giulio de’ Medici, 1478–1534), Berengario visited Rome a second time and stayed there for about 5 months during 1525–1526. The Pope had sent for Berengario to attend Cardinal Pompeo Colonna (1479–1532), who was suffering from a carcinoma. Berengario’s treatment of the cardinal was so successful that he left Rome enriched by a large fee and the gifts of art objects, including a beautiful painting entitled *St John in the wilderness* by Raphael (Raffaello Sanzio, 1483–1520), and some precious vases designed by the famous goldsmith Benvenuto Cellini (1500–1571). On his return to Bologna, Berengario lost his position as Lecturer in surgery and, for a still unknown reason, left the city in haste. In his *De morbo gallico* (The French Disease, referring to syphilis), the famous Italian anatomist Gabriele Falloppio (Fallopius, 1523–1562) imputes Berengario’s abrupt departure from Bologna to the fact that he had been charged with human vivisection: “this man so hated Spaniards that, when he was at Bologna, he took twin Spaniards suffering from syphilis and determined to practice vivisection on them: being ruined for this reason, he went to Ferrara” (Falloppio, 1564). This is an exaggeration from Falloppio, as there is no direct evidence supporting the accusation of human vivisection. What Berengario called *Anatomia vivorum* is nothing else than the so-called *Anatomia fortuita*, that is, what physicians can see during various surgical operations. In his *Commentaria*, Berengario is clear about that: “For in our time anatomy is not practiced on the living, unless, perhaps, by physicians as sometimes falls to my lot in cutting an abscess, etc., when they acquaint themselves with the anatomic relations of the organs (members), positions and operations and all the things that are requisite in anatomy” (Berengario da Carpi, 1521).

In any event, Berengario left Bologna around 1526 and went to Modena and then to Ferrara, where, by 1529, he had become court surgeon to Alfonso I d’Este (1476–1534), the son of Ercole, who succeeded his father as Duke of Ferrara. According to some contemporary accounts, Berengario died in Ferrara on November 24, 1530, and was buried there in the church of San Francisco (Guaitoli, 1880). However, the exact date of his death is still a matter of controversy and his remains in the church of San Francisco were never found. Berengario was a rich man when he died and his last will indicates that he had designated his nephew Damiano, the son of his brother Giovanni Andrea, as the general legatee of his estate. However, for reasons unknown to us, his property and estates were finally taken into the possession of the Duke Alphonso d’Este, the husband of Lucrezia Borgia (1480–1519), in defiance of Berengario’s last will and testament (Lind, 1975; Merlini et al., 2003; Prioreschi, 2007).

Let us now examine the major scientific contributions of this singular medical scholar of the Renaissance.

## Skull Surgery

**Figure 2:**
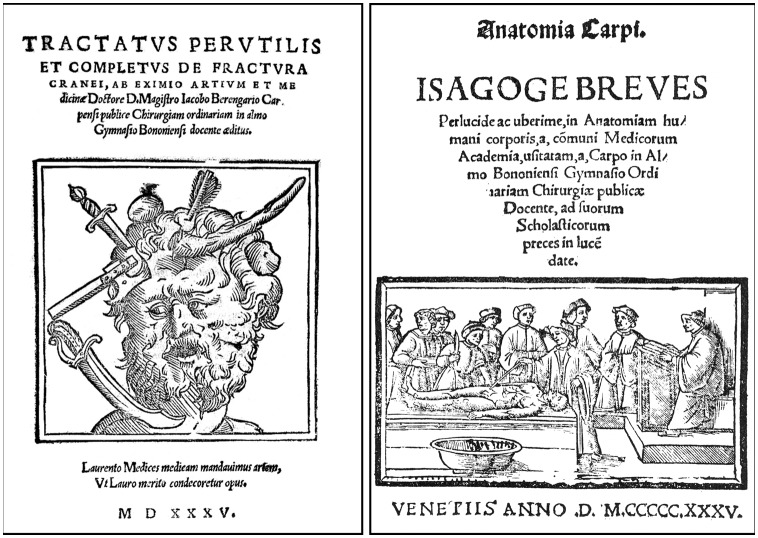
Frontispieces of the Venice 1535 editions of Berengario’s *De fractura cranei* (left), which was first published in Bologna in 1518, and of Berengarios’s *Isagogae breves* (right) that first appeared also in Bologna in 1522 (Courtesy of the National Library of Medicine). The typical medieval dissection scene displayed on the title page of the Venetian edition of *Isagogae breves* is at odds with Berengario’s own involvement in dissection and his insistence on the prominence of observation over authority in anatomy. The very same figure appeared in the frontispiece of other 16th century publications, including works by Galen and Avicenna.

In 1517, Berengario was called to Ancona, where Lorenzo II de’ Medici (1492–1519), Duke of Urbino, had been wounded in a battle on March 28, resulting in an occipital fracture and consequent shock trauma. Berengario used novel procedures to treat his reputable patient, who fully recovered from his wound. The ensuing dispute between Berengario and his colleagues over the way Lorenzo de’ Medici should have been attended to on the battle field prompted Berengario to write his famous treatise on the fractures of the skull (*Tractatus de fractura cranei*). Dedicated to Lorenzo de’ Medici, the book was first published in 1518 in Bologna (Berengario da Carpi, 1518), but went through several editions, including that of Venice in 1535 (Figure 2, left). Berengario’s *De fractura cranei* is a historical landmark work on skull surgery. It is the first book entirely devoted to head wounds and their surgical management. Besides a full description of Lorenzo de’ Medici’s wound, the volume contains several other cases of skull fractures that are thoroughly discussed based on Berengario’s own observations as well as those of his colleagues. While examining these various cases, Berengario pays close attention to the relation that exists between the precise location of the wounds and the resulting neurological symptoms. He also addressed prognosis, diagnosis and treatment, and provided detailed descriptions of various craniotomy procedures.

In its strikingly modern format, the book comprises a detailed description of the symptomatology of various patients who died as a result of head trauma and also includes accurately described subdural and epidural hematomas, *contrecoups* lesions, and what is known today as subdural empyema (Di Ieva et al., 2011). Berengario clearly distinguishes symptoms that occur when the meninges are involved and those in which they remain intact (Di Ieva et al., 2011; Dowling and Goodrich, 2016; Nanda et al., 2016). Furthermore, the symptomatology of patients with brain concussion is very detailed: redness of the eyes, bilious vomiting, loss of speech, impaired vision, vertigo, fever, vomiting, and falling down. This description is directly drawn from the encyclopedic surgery treatise (*Kitab al-Tasrif*) written around the year 1000 by the celebrated Arab physician Albucasis (936–1013; Spink and Lewis, 1973), whom Berengario cited no less than 20 times in his *De fractura cranei*. Berengario’s verbatim citation of Albucasis’ description of the symptomatology, which he refers to as *Prognostica Albucasis*, is as follows:

“Consider the signs and if you see among them some which are of particularly serious significance, such as vomiting of bile, loss of mind, cutting off of voice, syncope, acute fever, bulging reddish eyes, and similar sings, death will result from those in whom they occur in many dispositions. But if you see signs that are not alarming and you hope to save your patient, proceed with this cure”

*(Berengario da Carpi, 1518, *De fractura cranei*, 1518, Part II, Chap XIV, folio 39v)*.

The book contains eight wood engravings depicting various instruments used in head surgery, including an illustration of a trepanation drill (folio XCIr) that certain neurosurgeons consider the ancestor of the modern instrument (Di Ieva et al., 2011; Dowling and Goodrich, 2016). Here again Berengario recognizes his debt toward Albucasis, whose surgery treatise contains some of the very first depictions of trephine burs similar to the one illustrated in his *De fractura cranei* (Spink and Lewis, 1973).

Berengario’s *De fractura cranei* does not contribute significantly to our knowledge of the brain, a subject that Berengario has dealt with in his subsequent works (see below), but it is nevertheless considered as the first modern treatise strictly devoted to head injuries. It offered Renaissance surgeons a systematic review of the extant literature, detailed illustrations and explanations of various surgical instruments and techniques, instructions for skull fractures management, and suggestions for neurological examination and follow-up of patients suffering from head trauma.

## Anatomia Sensibilis

Berengario’s anatomical background was influenced by Mondino de’ Liuzzi (Mundinus, ca. 1270–1326), who held the chair of anatomy at the University of Bologna and was at the origin of the rebirth of human dissections in early Renaissance (Olry, 1997). Around 1316, Mondino wrote his famous *Anathomia*, a dissection manuscript destined to his students. Thanks to the discovery of printing around 1455, Mondino’s *Anathomia* became widely known and went through multiple re-editions in various European cities from 1478 to 1550 (Mondino de’ Liuzzi, 1478). Berengario himself published in Bologna a version of Mondino’s work in 1514 (Berengario da Carpi, 1514). However, his most influential work was *Commentaria super anatomiam Mundini* (Commentary on the anatomy of Mondino) that he published in Bologna in 1521 (Berengario da Carpi, 1521). A shorter (80 folios) and more affordable version of the book, which provides a concise and detailed description of the human anatomy, as well as a guide for dissection, was published a year later under the title *Isagogae breves* (A short introduction to human anatomy; Berengario da Carpi, 1522, 1523). The book went through several editions, including the one published in Venice in 1535 (Figure 2, right). In the present account, quotes are principally derived from the 1523 edition of the book because it is the only version that contains Berengario’s illustration of the brain (Berengario da Carpi, 1522, 1523). The American philologist Levi Robert Lind (1906–2008) has provided excellent English translations of Berengario’s *Isagogae breves* and *De fractura cranei* (Lind, 1959, 1990), and I have drawn from his work most of the excerpts listed below, but with some minor modifications based on my own reading of the original texts. I have also used the English translation of some sections of Berengario’s *Commentaria* by Cambridge medical historian Andrew Cunningham (Cunningham, 1997).

Although modestly titled, Berengario’s *Commentaria* is an extensive, 528 folios-long work in which the author gives detailed commentaries upon each short section of Mondino’s medieval text with criticisms and emendations based on what he had observed during his own dissections. The purpose for writing the book is clearly expressed in the following excerpt:

“When I saw so many and so great altercations between those writing on Anatomy, I resolved through means of a Commentary to draw together, by some quite brief summary, what I have seen by long experience in dissecting the bodies both of the living and the dead, and what I have sought in long reading. And my guide will be the excellent Mundinus of Bologna… In this exposition I will add some noteworthy things, not without their usefulness, from more recent writers, always with the senses and some authorities and reasonings of the divine Galen as my guide”

*(Berengario da Carpi, 1521, *Commentaria*, folio iiii r)*.

Throughout the book, Berengario argues in favor of the prominence of observation over authority in anatomy, and his motto appears to have been “One must not believe other authorities when experience and sense-perception run counter to them” (Berengario da Carpi, 1522, 1523). For Berengario the real proof in anatomical study can only come from the testimony of the senses, and this is what he called *anatomia sensibilis*, a procedure that he used for both investigation and teaching purposes (French, 1985; Mandressi, 2003).

Berengario’s remarkable knowledge of the scientific literature makes him unique among pre-Vesalian anatomists. Besides quoting all the usual Greek, Latin and Arabic authorities, including some who escaped the attention of other anatomists, he critically reviews the findings of Italian and foreign contemporaries. In addition, he comprehensively assessed the various anatomical terminologies as well as the merits of the translations of classic texts that he had at hands, thus making him the best philological anatomists of his time (Lind, 1975).

**Figure 3:**
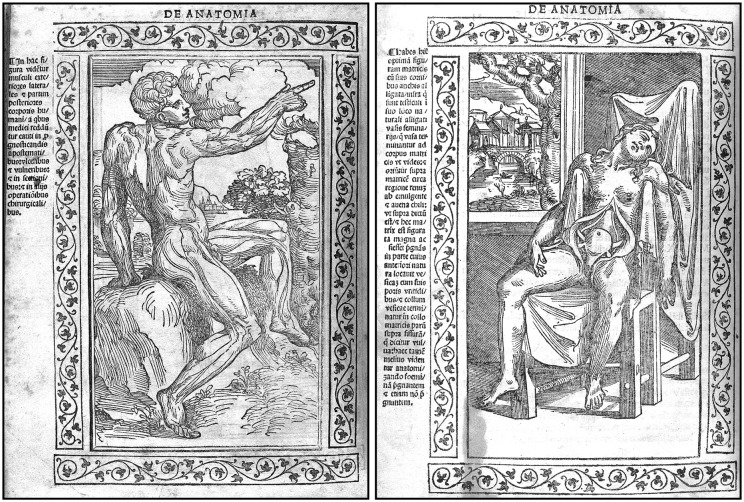
Two woodcuts from the 1523 edition of Berengario’s *Isagogae breves*. The figure on the left (folio 71v) shows a man standing in nature with his muscular anatomy particularly well outlined, whereas the figure on the right (folio 23v) depicts a woman sitting in a chair with abdominal viscera, particularly the uterus, clearly visible. The drawings are surrounded by floral motifs, and a detailed legend is provided on the left of each figure. These woodcuts might be the work of Hugo da Carpi (1455–1523; Courtesy of the National Library of Medicine).

Berengario was also the first anatomist to use illustrations based on direct observations to complement his text (Calkins et al., 1999; Dowling and Goodrich, 2016), initiating a fruitful collaboration between anatomists and artists that were to last long after the end of the Renaissance. Both the *Commentaria* and the *Isagogae breves* contain several illustrations directly drawn from nature and perfectly integrated within the text, in contrast to previous works in which a few rare figures inspired by medieval iconography were added afterward. The wood engravings that illustrate his texts show striking images of *écorchés* standing in beautiful landscapes that became archetypal figures in later periods (Figure 3). The figures that describe the complex arrangement of the abdominal muscles (11 of the 21 illustrations of Berengario’s *Commentaria* are devoted to myology) are considered a masterpiece of artistic grace and anatomical precision (Choulant, 1920; Calkins et al., 1999). They are believed to have inspired the paragon of Renaissance anatomy, Andreas Vesalius (1514–1564), in the preparation of his landmark treatise *De humani corporis fabrica* that came out in 1543 (Vesalius, 1543). Berengario’s figures, however, vary in excellence, and the best ones appeared to have been prepared for the use of artists rather than anatomists (Figure 3). These figures (plates 14–18 of the *Commentaria*) might have been the work of one of Berengario’s contemporaries, Hugo da Carpi (ca. 1486–1532), renowned for his stylistic contribution to the chiaroscuro woodcut (Choulant, 1920).

Besides his original contribution to the organization of muscles and the nervous system (see below), Berengario gave the first careful description of many anatomical structures of the human body, including the sphenoid sinuses, the vermiform appendix, the tympanum, the arytenoid cartilages, the thymus gland, the seminal vesicles, the pancreas, the kidneys, the pineal gland and its position in regard to the third ventricle of the brain. He also provided the first comprehensive description of the action of the cardiac valves and the vocal organs. The injection of fluids (tepid water, principally) in the vasculature of the kidney and other organs to study their functional arrangement is a striking demonstration of the novelty and exploratory aspects of Berengario’s method of anatomical research (De Santo et al., 1999).

## Brain Anatomy

Berengario has shed a new light on the overall anatomical organization of the human brain and cranial nerves while providing one of the first realistic pictures of this organ that he saw as the major coordinator of cognition. His meticulous dissections of more than one hundred human heads, as well as that of many different animal species, allowed him to discover many new aspects of brain arrangement that he reported in detail in his *Commentaria* and, more succinctly, in his *Isagogae breves*. We shall now briefly review his contributions to some features of the brain and associated structures to which he paid a particularly close attention.

### The *Rete Mirabile*

**Figure 4:**
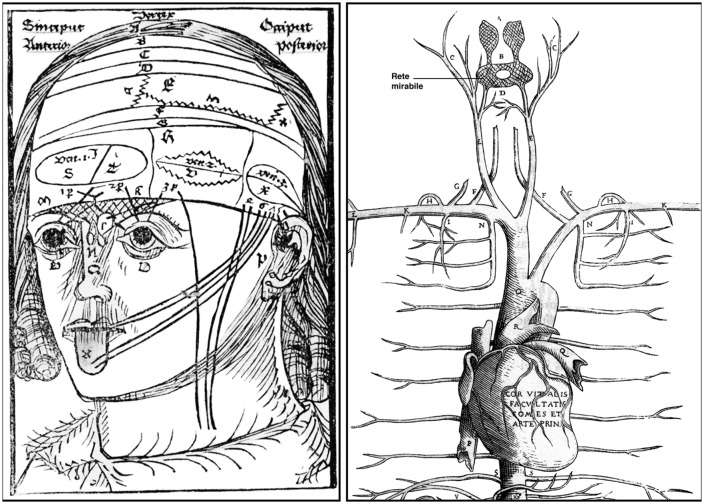
Left: a woodcut from Magnus Hundt’s *Anthropologium* published in Leipzig in 1501. It offers a crude, schematic view of a human head with layers of the scalp (labeled a, b, c, d), bones, meninges and brain, cranial nerves, ventricles (the three major cells) and the *rete mirabile*. The latter (shown here for the very first time) is depicted as a roughly triangular crisscrossed structure that extends from the bridge of the nose over both eyes to the lower part of the forehead. Right: detailed of a woodcut from Andreas Vesalius’s *Tabulae Anatomicae Sex* (Tabula III) published in Venice in 1538. The illustration shows the upper arterial vascularization and includes a clear depiction of the *rete mirabile* (the name has been added to the original drawing to facilitate its reading). Vesalius’s letter B indicates the *rete mirabile* proper (*plexus reticularis*), whereas the letter A points to the *plexus choriformis*, a structure that Vesalius saw as an upper extension of the *rete mirabile* that plunges within the anterior (lateral) ventricle (Courtesy of the National Library of Medicine).

**Figure 5:**
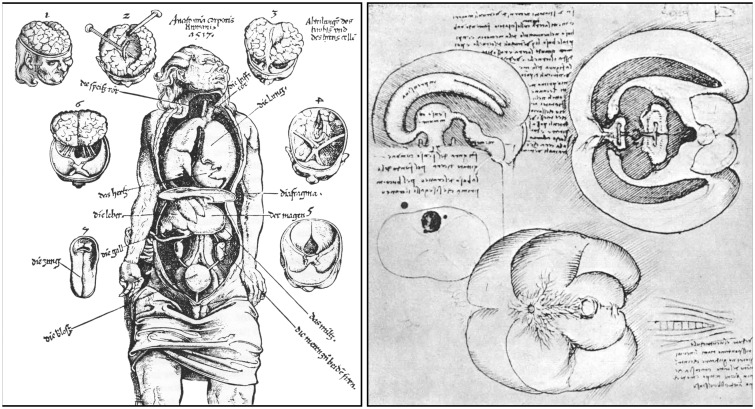
Left: a woodcut included in Phryesen’s *Spiegel der Arzney* that appeared in Strasburg in 1518. The figure (folio 9v) displays a human body down to the knee, with the thoracic and abdominal cavities cut open, and surrounded by small figures representing six different stages of a human brain dissection. The names of the parts are given mostly in German. Right: details from a figure (plate QC7r) drawn by Leonardo da Vinci around 1504–1507. The sketch in the upper left portion of the figure illustrates the ventricular system, as visualized following wax injection in the cerebral ventricles of an ox. The drawing at the bottom of the figure depicts the base of a human brain covered, in part, by a spidery structure corresponding to the *rete mirabile* (from Parent, 2009).

One of Berengario’s major concerns was the existence of the so-called *rete mirabile* (admirable network), a complex vascular plexus that was thought to be present at the basis of the human brain, exactly where the arterial circle of Willis is located. Galen, who attributed the discovery of this vascular network to Herophilus of Alexandria (ca. 335–280 BC), believed that the *rete mirabile* served to transform the vital spirit (*spiritus vitalis*) from the blood into the psychic spirit (*spiritus animalis*) that was stored into the brain ventricles, where it controlled the major cognitive functions of the brain. According to Galen, the animal spirit exerted its motor and sensory functions by flowing into the peripheral nerves that he described as hollow tubes (Clarke and Dewhurst, 1996; Clarke and O’Malley, 1996). Although based on dissections of various animal species, excluding human bodies, the Galenic concept of the *rete mirabile* remained intact for more than a millennium (de Gutiérrez-Mahoney and Schechter, 1972; Pranghoffer, 2009). Its existence was even emphasized by medieval anatomists, including Mondino, who provided detailed instructions for its dissection in his 1316 *Anathomia*. The enigmatic network has also been illustrated by several Renaissance anatomists and artists. For example, the German physician and philosopher Magnus Hundt (1449–1519) has portrayed the structure in a highly schematic manner in his 1501 treatise *Antropologium* (Hundt, 1501). A somewhat more “realistic” version of the vascular plexus was provided by the illustrious painter, sculptor and engineer Leonardo da Vinci (1452–1519), who was actively dissecting human bodies during the years 1504–1507 (Figures 4, 5).

Like all medieval and renaissance anatomists, Berengario shared most of Galen’s views. He was nevertheless the first to shed doubt on the existence of the *rete mirabile* (Russell, 2013). His disbelief is first expressed with some reservations in his *Commentaria*.

“Note, reader, that I have worked hard to discover this *rete* and its location. I have dissected more than a hundred heads almost solely for the sake of this *rete* and even now I don’t understand it. It is true that posteriorly under the dura mater, at the right and left of the gland [pituitary] beneath the lacuna [infundibulum of the third ventricle] of Mondino, I often saw and touched something like an intricate network that might be judged to be the *retemirabile*. […] I found a complex as aforesaid, but I do not know whether this is that *rete* or another complex. If there is a *rete mirabile* such as Galen mentions as fact, I believe that the aforesaid complexes are the *rete mirabile*, but since Galen said earlier that the *rete mirabile* is, at least in part, in the substance of the dura mater, I believe that the whole *rete mirabile* could not be seen because the dura mater would be so intricately involved with it that it would not be possible to distinguish one from the other. […] I have careful eyes, hands, and instruments (diligentem oculum and mantú and instrumenta) suited to separating the dura mater from the cranium, and I have dissected many heads, as I said above, and did not find such a *rete* except in that place mentioned by me. It is my opinion that if there is a *rete* there in the latter location, it must be concluded that Galen erred, because he says that when the ascending arteries are above the base of the skull, immediately they are divided very minutely and form the *rete*; then he says that from all the branches of the rete again two branches of arteries are formed that perforate the dura mater and ascend to the brain. This, however, is not true, because many times I have inserted a little stylus (styllum) above the dura mater into the aforesaid large ascending branches which are near the optic nerves [internal carotids], and I have found that the stylus penetrates directly downwards through those arteries without any obstacle as far as the base of the skull; and if the aforesaid arteries were so reticulated above the base of the skull and divided very minutely, as Galen says, the stylus would be unable to penetrate downwards through them to the bone because it would find the *rete* to be an obstacle. […] Thus I believe that Galen imagined the *rete mirabile* but never saw it, and I believe that all others after Galen that spoke of the *rete mirabile* did so on the strength of his opinion rather than their own perception of it”

*(Berengario da Carpi, 1521, *Commentaria*, 1521, folio CCCCLIX r/v)*.

Berengario is even more categoric in denying the existence of the *rete mirabile* in man in the 1535 version of *Isagogea breves*, where he clearly mentioned not having been able to see that network. However, even though he denied the existence of a human *rete mirabile*, Berengario readily accepted Galen’s views on the animal spirit; he simply displaced the locus of production of this humor from the *rete mirabile* to the blood vessels of the pia mater, as revealed in the following excerpt.

“I have never seen that network. I believe that nature does not perform through many parts what she can achieve through few, and that she is able to refine this spirit in the very minute branches of the arteries ascending above the dura mater attached to the base of the skull and continuing through the pia mater as far as the center of the brain. Therefore, this network does not exist in that place between the dura mater and the base of the skull. I have given many other reasons for this in the *Commentaria* on Mondino to which for the sake of brevity I refer the readers. Among the other reasons I am influenced by sensory experience”

*(Berengario da Carpi, 1535, *Isagogea breves*, 1535, folio 49v)*.

Despite Berengario’s convincing argument for the non-existence of the *rete mirabile* in man, Galen’s influence upon the medical thought was so powerful that many Renaissance anatomists refused to abandon the concept and even continued to illustrate the structure. This is the case of the German anatomist Johann (Eichmann) Dryander (1500–1560) who, 16 years after Berengario’s *Commentaria*, published a treatise on the dissection of the human head that contains a schematic drawing of the human brain similar to the one produced earlier by Hundt (Figure 4, left) and in which the *rete mirabile* is clearly delineated (Dryander, 1537). One year later, Vesalius himself depicted this enigmatic structure in one of his *Tabulae anatomicae sex* (Six anatomical tables) published in Venice (Vesalius, 1538). This illustration (Figure 4, right) sketches the internal carotid merging into a circular net that Vesalius described as follows: “*plexus reticularis*, at the base of the brain. The *rete mirabile* in which the vital spirit is elaborated into animal spirit.” Obviously not yet liberated from Galen’s teachings, Vesalius depicts the *rete mirabile* as a large netlike structure endowed with two bulbous extensions that ascend directly within the lateral ventricles (Figure 4, right). However, in his milestone treatise *De humani corporis fabrica* published 5 years later, Vesalius denied categorically the existence of the *rete mirabile* (Russell, 2013). Without referring to Berengario’s work, Vesalius admitted that during his public lectures he had to use animals such as sheep or oxen to reveal a network that does not exist in human (Vesalius, 1543). He took credit for the demonstration of the non-existence of the *rete mirabile* and treated harshly the anatomists who, before him, believed in a structure that they had never seen, but described only on the basis of Galen’s writing. All prominent anatomists who came after Vesalius either denied the existence of the human *rete mirabile* or mentioned that this structure is insignificant in man.

### Realistic Depictions of the Human Brain

Based on Galen’s view of the ventricular localization of brain functions, medieval scholars in both Europe and the Islamic world developed the ventricular or cell model of the localization of mental faculties. This model implies that inputs from the five external senses converge towards the anterior ventricle (the seat of the common sense), that this information is then conveyed to the middle ventricle, where its significance is interpreted (the seat of cogitation or cognition), and that the decoded information is finally stored within the third ventricle (the seat of memory; Clarke and Dewhurst, 1996; Green, 2003). This concept, which pervaded the entire Middle Ages and a large part of the Renaissance, has led to a highly schematic depiction of the human brain, where little attention is given to anatomical details and emphasis placed on the ventricular cavities displayed in the form of three large and often interconnected circles (Figure 4, left).

The first realistic representation of the human brain appeared in a vernacular medical treatise entitled *Spiegel der Artzny* (Mirror of medical art) published in Strasburg in 1518 by Lorenz Fries, a Dutch physician best known under his Latin name Laurentius Phryesen (ca. 1480–1532; Phryesen, 1518). It shows the brain in the form of six different images scattered around the cadaver of a man whose thoracic and abdominal viscera are displayed. The drawings, numbered 1–6, depict six different stages of a human brain dissection (Figure 5, left). This woodcut has been attributed to the German artist Johann Wechtlin (fl. 1502–1526), who drawn it from a dissection performed in Strasburg in 1517 by Wendelin Hock (fl. 1513–1535), a German surgeon from Brackenau in Württenberg. Despite its crudeness, the overall display is original, and the depiction of the brain in the form of six small figures surrounding the cadaver is new and unique (Choulant, 1920). The plate bears the inscription *Anatomia corporis Humani, 1517*, and was first published by Johann Schott (1477–1548) of Strasburg in 1517 as an anatomical fugitive sheet (fliegende Blätter) associated with a book by Hans von Gersdorff (ca. 1455–1529) entitled *Feldtbuch der Wundartzney* (Fieldbook of surgery; von Gersdorff, 1517).

Leonardo da Vinci also provided some quite accurate depictions of the basis of the human brain, together with a sagittal and a transversal view that he used principally to illustrate the lateral ventricles, as will be discussed below (Figure 5, right). Unfortunately, Leonardo’s figures were never published. They were known only to some of his close friends, so that they did not contribute, as they should have, to the development of brain iconography.

**Figure 6:**
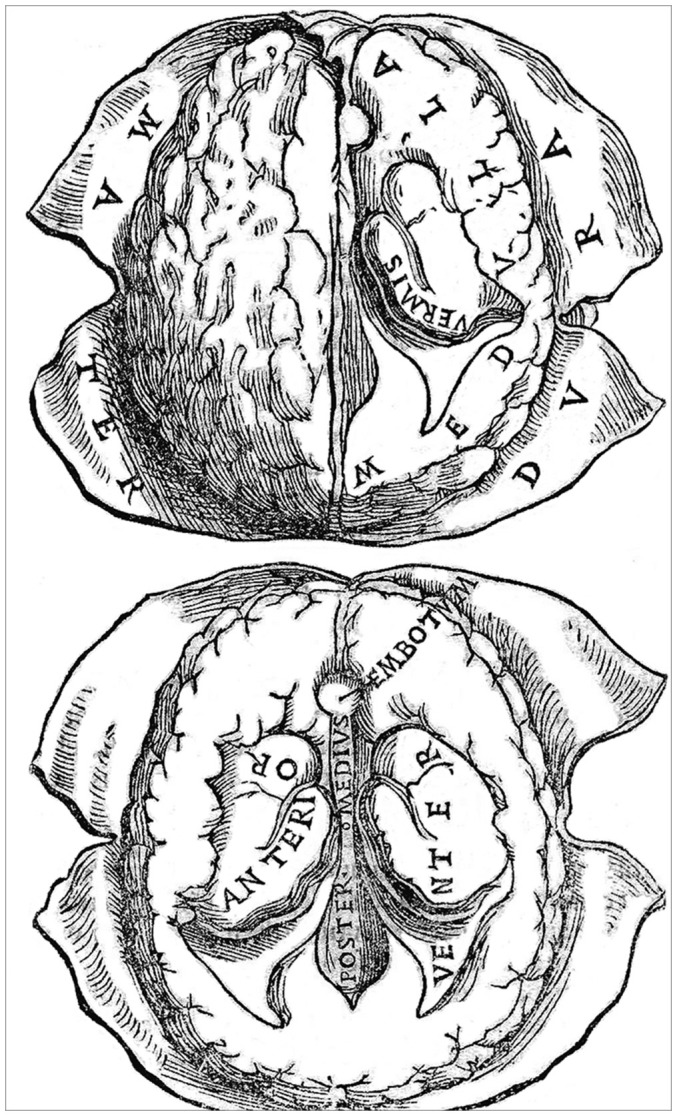
A woodcut (folio 56r) from the Bologna 1523 edition of Berengarios’s *Isagogae breves*. It offers two fairly accurate horizontal views of the human brain drawn from nature, with emphasis on the organization of the meninges, particularly the dura mater, and the ventricular system. The top figure, which depicts the brain at a slightly more dorsal level than the bottom figure, shows the left hemisphere intact, with indications of sulci and gyri, whereas the right hemisphere has been dissected out so as to show the dorsal aspect of the lateral ventricle and its prominent *vermis* (choroid plexus). The bottom figure provides a more detailed view of the ventricular system on both sides of the brain. The anterior (lateral) ventricle is shown along its full anterior-posterior extent, with the middle (third) and posterior (fourth) ventricles depicted at a lower level. The middle ventricle is bordered anteriorly by the *embotum* (infundibulum and pituitary stalk). Courtesy of the National Library of Medicine.

Berengario also provided a representation of the human brain drawn according to nature. It appeared in the 1523 edition of his *Isagogae breves*, which, unlike the 1522 edition, featured four illustrations of the heart and two of the brain, with some variations in the woodcuts showing the muscles. Berengario’s figures are far more superior and much closer to nature than the illustration provided by Phryesen. The woodcut offers two images of the human brain seen from above (Figure 6), with a faithful depiction of the ventricular system that contrasted markedly with medieval renditions that were current at that time (Figure 4, left). The upper picture shows a brain that is intact, whereas that at the bottom represents a more ventral plane of dissection allowing a clearer view of the ventricular system. The position and relative importance of the lateral ventricles in respect to the third and fourth ventricles, referred to here as the middle and posterior ventricle, respectively, are also represented. In regard to these figures, Berengario states “I have accommodated such figures of the brain as I was able, in which some of the matters previously described can be understood, as you see them” (Berengario da Carpi, 1522, 1523). This sentence reinforces the idea that Berengario was a pioneer in recognizing the usefulness of illustrations to enhance understanding of verbal descriptions in anatomy (Clarke and Dewhurst, 1996; Russell, 2013). Although Berengario’s figures do not have the quality of execution of those that will appear later in Vesalius’ *Fabrica*, they nevertheless can be considered as the first accurate description of the human brain. From that point of view, it can be said that Berengario paved the way for Vesalius or, as Falloppio put it in his *Observationes anatomicae*: “what Carpi had begun Vesalius perfected” (Falloppio, 1561).

### Meninges and Cranial Nerves

In his account of the anatomy of the human body, Berengario follows Mondino in dividing the body into three major cavities (*venter inferior*, *venter medius* and *venter superior*) and in describing all the “members” (organs) contained within each cavity. For each organ he systematically lists the main facts about its substance, size, shape, location, connection, and complexion: a typical scholastic procedure. His carefully recorded connections for members—liver by veins, heart by arteries, and brain by nerves—is a consistent attempt to place the specific organ in the overall organization of the body and it has some physiological and even pathological implications to which he alluded to in the short summary placed at the end of each organ account. His description of the meninges and cranial nerves follows closely this procedure.

After having given specific technical details for the optimal visualization of the brain coverings, Berengario provided a thorough description of the intimate fabric, topographical distribution and vascularization of the dura mater and the pia mater, the only two brain membranes known during his time (see folio 53 r/v of the 1523 edition of *Isagogae breves*). He referred to the dura mater as follows: “This is a quite thick, tendinous and strong panniculus, also porous, so that the vapors may escape from the brain. Its form is flat, extended in a circular manner, embracing all the medulla [brain substance] within itself together with the pia mater of the brain.” The closing statement is as follows: “Its size, site, connection, and complexion are evident. It is a single panniculus. Its service, beyond the services described, is to clothe the brain with the pia mater along its length, width, and depth by surrounding it and penetrating it has explained above. It helps also by mediating between the hard bone and the quite soft pia mater. It also assists by sustaining the veins which nourish the brain and members near it. It suffers all kinds of ills. Its severe injury is dangerous” (Berengario da Carpi, 1522, 1523).

His description of the pia mater is more succinct, but Berengario takes this opportunity to explain his view about the role of this membrane in the generation of the animal spirit (psychic pneuma), in lieu and place of the *rete mirabile*: “Under this dura mater is another thin membrane with very slender arteries and veins spread like a net throughout it and immediately attached to the brain. It is called the pia mater and the *secundina* [chorion] because it nourishes the brain as the *secundina* nourishes the fetus. In my judgment it is in these very small branches of the arteries dispersed everywhere in the pia mater that the blood or vital spirit is rendered subtle and prepared so that in the substance of the brain and in its ventricles it may be made into animal spirit, as I have said in my *Commentary on Mundinus*” (Berengario da Carpi, 1522, 1523).

In the section devoted to cranial nerves (see folios 56v and 57r of the 1523 edition of *Isagogae breves*), Berengario follows his mentor Mondino (and Galen before him) in describing seven pairs instead of 12, as in the current usage. For Berengario, “Nerve is neuron in Greek.” It is a consimilar member [an organ of the same shape and substance throughout] of white viscous substance, oblong and round in form, tenacious and hard to separate.” In accordance with the concept promoted by the Alexandrian school of medicine and transmitted through Galen and Mondino, Berengario considers nerves as organs of sense and motion, the sensory nerves being softer than the motor nerves. His description of the cranial nerves is, as usual, detailed, lively, and offers some new and challenging concepts, as revealed by his depiction of the olfactory and optic nerves.

He portrays the olfactory nerve, which Galen does not consider in his classification, as follows: “oblong substances, one on each side, adhering to the pia mater. Their heads are somewhat thick. These are called by many the mamillary *carunculae* [olfactory bulbs]; they are instruments of the sense of smell and Galen does not call them nerves, for they are soft. In their direction the panniculi of the brain and the bone of the forehead are perforated like a sieve [the lamina cribrosa of the ethmoid bone], both for odors as well as for purging the superfluities of the brain when necessary, although they are for the most part purged by way of the *colatorium*, which is in the direction of the aforesaid *embotum*” (Berengario da Carpi, 1522, 1523).

As for the optic nerve (the first pair in Galen’s classification), Berengario describes them as follows: “After these *carunculae* you will see two larger nerves which serve the eyes for vision. These are seen to cross [optic chiasma]; concerning this point there is still no agreement. And these are called the optic nerves or of vision; according to some they are concave or perforated; this, however, is not visible in the dead creature.” The latter sentence is of importance as it contradicts the hollowness (or perforation) of the optic nerve, which was a central tenet in Galen’s conception of the diffusion of the animal spirit from the cerebral ventricles to the periphery. Berengario also underlined the controversy that existed since the Antiquity about the partial or total decussation of the optic nerve fibers at the level of the optic chiasma and the role of such a crossing in vision.

Then comes a description of the trajectory and function of the other pairs of cranial nerves that follows Galen’s description. The second pair (our oculomotor nerve) is said to “gives motion to the eyes.” Our trochlear nerve is described as being part of the second pair, whereas no mention is made of the abducens nerve. The third and fourth pairs correspond approximately to the sensory and motor roots of our trigeminal nerve, respectively. The fifth pair comprises both our facial and vestibulo-cochlear nerves, but Berengario uses functional and topographical criteria to subdivide it into two distinct parts, one endowed with branches “that spreads out to the face” and another with branches that course “in the direction of the ear and serves the sense of hearing.” Berengario’s sixth pair corresponds to a mix of our glossopharyngeal, vagus and spinal accessory nerves; part of it is described as being associated topographically and functionally with the third pair. Finally, the seventh pair (our hypoglossal nerve) is depicted as a nerve that “gives motion to the tongue and also to some muscles of the epiglottis and also gives the sense of taste to the tongue itself.”

Galen’s seven-pair classification of cranial nerves will be slightly modified in the 17th century by the Oxford physician and anatomist Thomas Willis (1621–1675). In his celebrated *Cerebri anatome* (Brain Anatomy), published in 1664, Willis identifies nine cranial nerves instead of seven (Willis, 1664). His seventh pair is a merging of our facial and vestibulo-cochlear nerves, his eighth pair a combination of our glossopharyngeal, vagus and accessory nerves, but he also recognizes a separate accessory nerve (Flamm, 1967; Pearce, 2017). It is only in the late 18th century that our present classification of 12 cranial nerves will be proposed, thanks to the work of the Prussian anatomist and polymath Samuel Thomas von Soemmerring (1755–1830), who was then a doctoral student (Pearce, 2017). This novel classification is thoroughly described in his doctoral thesis entitled *Anatomica de basi encephali* (Anatomy of the base of the brain) published in Göttingen in 1778 (Soemmerring, 1778).

### Brain Medulla and Ventricular System

Berengario’s description of the various brain structures and of the ventricular system is to be found in folios 54 r/v and 55 r/v of the 1523 edition of his *Isagogae breves*. During Berengario’s time, the brain tissue was referred to as the *medulla of the brain*. However, Berengario considered this expression improper “because it [the brain substance] neither nourishes nor moistens the bones near it as the medullae of other bones do. But the bones of the head are nourished so that they may preserve their medulla.” In accordance with Galen’s teachings, he described the brain matter as “conspicuous, softer (*mollior*) in front and above than behind and below. Its size exceeds that of the brain in other animals both on account of its abundance of the animal spirit as well as on account of the fact that by its cold and humid complexion (*cōlexione frigida and humida*), and in accordance with reason, it tempers these spirits, which come very hot from the heart.” He noted, the presence of gyri and sulci on the brain surface: “It has many folds (*multas plicaturas*) that are visible at first sight and also many hidden folds which are seen in its dissection.” However, he illustrated them in a rather sketchy manner (Figure 6). Berengario emphasized the fact the human brain forms a single organ occupying the entire cranial cavity. He described it as being composed of a large and smooth anterior part and a small and hard posterior part “called cerebellum by Aristotle.” He recognized that the anterior part (the *cerebrum*) is divided along its entire anterior-posterior extent into two halves (the cerebral hemispheres) by a large meningeal septum (the *falx cerebri*), “so that its substance and its ventricles may be distinct and double.”

After having portrayed grossly the medulla of the brain, Berengario undertakes a much finer and meticulous depiction of the ventricular system. The medieval three-circles model is here replaced by a realistic depiction of the ventricular system, with emphasis on the remarkable anterior-posterior extension of anterior (lateral) ventricle (Figure 6). He also noted the presence of choroid plexuses in all ventricles and pointed to a distinct foramen that links the anterior and middle (third) ventricles (the foramen of Monro). Berengario also correctly interpreted the trajectory of the cerebral aqueduct leading to the posterior (fourth) ventricle and the topographical relationship between this ventricular conduit and the pineal gland. The following excerpts are particularly revealing of his findings.

“On each side of this duplication [the two hemispheres] you will find one notable vacuity called a ventricle which is stretched out lengthwise and somewhat obliquely descending laterally toward the rear [inferior horn of the lateral ventricle]. […] The substance of the brain divides these ventricles so that if injury occurs in one, it may not occur in the other. The operations of one part of these ventricles are similar to the operations of the ventricle equal to it. In the ventricle on both sides near the base is a pellicular red substance called a worm [*vermis* or choroid plexus], composed of veins and arteries that extend from one end to the other in each ventricle. This has motion, according to some, opening and closing the ventricles voluntarily. […] Having seen the foregoing, remove the notable part of the medulla of the brain so that one may carefully see the other vacuities of the brain, noting in the anterior base of the aforesaid two vacuities a foramen that is common to them [foramen of Monro]. Through this foramen the spirit and some humidities contained in them descend and pass out to a certain vacuity stretching toward the basilar bone near the place where there is a certain glandulous flesh [pituitary gland] under the crossing of the optic nerves. This vacuity is called *lacuna* by Mundinus, *head of the rose* by Avicenna (*ab Avic. caput rose*), and *embotum* by others because it is broad above, narrow below, and surrounded on all sides by a thin panniculus as far as the basilar bone [infundibulum and pituitary stalk]. […] In the posterior part of this middle ventricle is a little foramen that reaches to another vacuity that descends toward the place where there is the beginning of the nape of the neck [aqueduct of Sylvius]. This vacuity is not in the cerebellum aforesaid, as many think, nor is it everywhere surrounded by the medullar substance of the brain, but it is situated between the posterior and anterior brain, notably surrounded toward the cerebellum by the pia mater which covers it [choroid plexus of the fourth ventricle]. Between this last vacuity and the middle ventricle aforesaid is a certain glandulous flesh [pineal gland] called *coronarium* because it is in the form of a cone or pineapple. This gland in that place sustains many veins of the pia mater ascending toward the center of the brain to nourish it”

*(Berengario da Carpi, 1522, 1523, *Isagogae breves*, 1523, folios 54v and 55)*.

Surprisingly, Berengario also made a clear allusion to what appears to be the head of the caudate nucleus protruding within the lateral ventricle: “Below the worms at their sides is a certain eminent part of the brain (*est certa pars cerebri eminens*) that many compared to the human buttocks (*natibus* *humanis*) in its form. This part both in elongation and closing of the ventricles touches its two portions together and separates them in the shortening and dilation of the ventricles” (*Isagogae breves*, folio 54v). Galen was one of the first anatomists to pay attention to the large masses of nervous tissue lying at the basis of the lateral ventricle, which he referred to as the gluteal parts of the brain. In some of his treatises he used the Greek term *glutia* (or buttocks) to describe these structures, whereas in others he compared them to human thighs (Parent, 2012). It is difficult to know precisely what Galen had in mind when he used these terms, but his awkward terminology was employed in the Medieval and early Renaissance periods, as mentioned by Berengario in his *Isagogea breves*. In the middle of the 17th century, Thomas Willis noted the striate appearance of this large mass of nervous tissue and, accordingly, named it *corpus striatum* (or chamfered body). He realized that this structure, to which he assigned a role in the control of motor behavior, comprises an intra-ventricular portion (our caudate nucleus) and an extra-ventricular portion (our lentiform nucleus), but did not specifically name these two components of the corpus striatum (Willis, 1664). The first accurate depiction of the caudate nucleus, as well as that of most of the other basal ganglia components, emerged early in the 19th century from the work the great German neuroanatomist Karl Friedrich Burdach (1776–1847). Burdach used the word *Streifenhügel* (streaked hillocks) to designate the structure, but he gave credit to the Italian anatomist Vincenzo Malacarne (1744–1816) for having been the first to mention the existence of such an elongated mass of gray substance lying along the base of the lateral ventricles (Burdach, 1819). However, Malacarne had most probably picked it up in a translation of Galen’s work by Hunayn ibn Ishaq (ca. 809–873 AD) early in the Middle Ages (Parent, 2012).

In his *Isagogea breves* Berengario does not comment on the presence of fluid into the ventricles, but he did so earlier in his *Commentaria*, where one finds the following sentence: “In my opinion, the gross superfluidity of the brain [watery excrement] can be seen in the majority of heads dissected because some of it is always in the ventricles of the brain. I believe that there is more of this moisture in one body than in others” (folio CCCCXXXIX). When Berengario wrote his detailed description of the ventricular system, he was probably unaware that, a few years earlier, his illustrious fellow countryman, Leonardo da Vinci, had succeeded in producing a cast of the cerebral ventricles. During the years 1504–1507, Leonardo injected hot wax into the cerebral ventricles of an ox and, after peeling off the brain matter, obtained a faithful model of the ventricular system (Clarke and Dewhurst, 1996; Parent, 2009). His direct transposition of the image of the ox cerebral ventricles onto one of his sketches of the human brain, although a scientifically questionable operation, nevertheless led to the very first realistic depiction of the ventricular system (Figure 5, right). Unfortunately, since Leonardo’s drawings remained unknown until they were rediscovered in the 19th century, they did not contribute to the advancement of our knowledge of brain anatomy during the Renaissance.

In contrast, Berengario’s studies opened the path to a reappraisal of the overall anatomical arrangement of the human brain. Yet, Berengario’s thoughts about the functional organization of this master organ were not significantly different from that of Leonardo before him and Vesalius after him: they all remained within the confines of Galen’s teachings. Berengario espoused the idea of the ventricular localization of higher brain functions. However, in contrast to the scholars of his time, who considered the anterior, middle and posterior ventricles as the seat of common sense, cogitation and memory, respectively, Berengario displaced these three major functions to the sole anterior ventricle. Hence, he believed that the complex mental operations underlying perception, interpretation and storage of information were sequentially executed in the voluminous lateral ventricle of the cerebral hemisphere. Such a view indicates that, despite his efforts to create a novel brain anatomy, Berengario, like most Renaissance medical scholars, was unable to totally free himself from Galen’s influence.

## Pre-vesalian Anatomists

Berengario da Carpi was born in the right place at the right time. He lived in one of the most exciting periods of European history: a time in which humans experienced a striking expansion of their geographical environment, with the discovery of new worlds, and a marked enrichment of their mind as a result of major achievements in the fields of sciences, arts, literature and philosophy. In the domain of human anatomy, the epithet “pre-Vesalian” is often attached to this unique period that extends from 1490, when Girolamo Manfredi (1430–1493) of Bologna published his *Anothomia—*a compendium of short anatomy texts of ancient authors (Singer, 1917) still embedded in astrology, chiromancy and physiognomy—to 1543, the year of the coming out of Vesalius’ *Fabrica*. Berengario was one of the most preeminent figures of the first contingent of pre-Vesalian anatomists, which also included Allessandro Achillini (1463–1512), Allessandro Benedetti (ca. 1450–1512) and Gabriele Zerbi (1445–1505). Most of these precursors were grounded in medieval scholasticism and used Mondino’s *Anathomia* as their guide. Their work was completed by a second contingent of pre-Vesalian anatomists, which comprised Andrés de Laguna (1499–1560), Niccolò Massa (1499–1569), Johannes Guenther of Andernach (1505–151574) and Johannes Dryander (1500–1560). We shall now briefly comment on the scientific contribution of each of these anatomists, their relationship with Berengario, and their effort to improve anatomical nomenclature.

Allessandro Achillini was born in Bologna where he taught philosophy and medicine. Most renowned for his philosophical contributions, he nevertheless wrote *Annotationes anatomicae* (Anatomical notes), a medicine book that was published posthumously (Achillini, 1520), but which records observations that he made between 1502 and 1506. The Latin style of this series of ill-arranged notebooks is highly confusing and at time difficult to decipher. The work nevertheless contains some interesting observations, such as a reference to the duct of the submaxillary gland (Wharton duct) and a description of the brain that is quite accurate for the time, with emphasis on the ventricular system, the fornix and the infundibulum. A native of the region of Verona, Alessandro Benedetti was successively an active physician in Greece (Crete), a successful professor of medicine at Padua and an excellent military surgeon in the Venetian army. His *Anatomice* (Benedetti, 1502) is the first Renaissance medical treatises giving priority to the Greek anatomical nomenclature over the Latino-Arabic terminology. Benedetti’s description of the brain and nerves (*Anatomice*, Book IV, chapters IX to XIII, folios 38–46) is tainted by Aristotelian theories and humoral concepts, with little useful anatomical insights. I find the first sentence of the chapters devoted to the brain particularly revealing of the flavor of the entire treatise: “The neck sustains the head, in which is contained the brain (*cerebrum viscerum*), the most excellent of the members and the nearest to the sky (*Anatomice*, Book IV, chapter II, folio 38v). After Beregario, Gabriele Zerbi was the second most important figure of the first wave of pre-Vesalian anatomists. Born in Verona he taught medicine and logic at Bologna and Padua and died under torture by the Turks. His *Anathomie corporis humani* (Anatomy of the human body) is the most complete and scholarly work entirely devoted to anatomy up to that year (Zerbi, 1502). The treatise is somewhat difficult to read principally because of the repetitive used of the scholastic method for describing each member of the body and the Aristotelian fashion of dividing the human body into anterior, posterior and lateral parts, in contrast to Mondino’s three main cavities. His description of the human brain (*Anathomie*, folios 111r to 118v) does not significantly improve that of Achillini.

The information regarding the academic and personal relationships between these pre-Vesalian anatomists is scanty, but there is some evidence that Berengario, Zerbi and Achillini worked together at Bologna sometime during the last quarter of the 15th century: Berengario appears to have studied under Professor Zerbi, while dissecting with his colleague Achillini (Lind, 1975). For unknown reasons, the relationship between Berengario and Zerbi appears to have been tumultuous, as reflected by the numerous harsh comments about Zerbi’s work and personal life that occurred in Berengario’s *Commentaria*. In the latter work, Zerbi’s name appeared on a total of 123 folios, and most of these quotes are mostly to correct, but sometimes to or agree, with Zerbi on various anatomical issues. At some point in the text, however, Berengario’s vindictive character took over and led him to make a vicious attack on the moral character of Zerbi himself, but also on that of two of his sons who were hanged in Rome as thieves. Such brawny remarks are typical of Berengario’s bad temper as well as of the vigorous and acrimonious debates that made the study of anatomy so exciting in the Renaissance, a period that saw the birth of numerous novel ideologies that were clashing with each other and with the well-implanted medieval philosophies. In his *Commentaria*, Berengario was more receptive to the wok of his former colleague Achillini than to that of is one-time Professor Zerbi, but he quoted Achillini only three times and, at one point (folio 494 v), he refers to Achillini’s work as *confusa anatomia*…

Mondino’s *Anathomia* was the basis upon which the pre-Vesalian anatomy was built. In contrast to the novelty of its content, which was an inspiration to the Renaissance anatomists, the form of this medieval treatise has posed them serious problems of interpretation. The text is written in a debased Latin and is cluttered with corrupted terms borrowed from Arabic, Hebrew, Greek and Latin. Even worse, several terms are used to designate the same anatomical parts, whereas the same name is often employed to single out several parts. Hence, pre-Vesalian anatomists progressively abandoned their search for the harmonization of Arabs and medieval scholastic authorities in favor a more practical approach, which consisted of substituting Greek for Arabic and Hebraic terms in the hope to generate a more coherent anatomical nomenclature. Guided essentially by Latin translations of Arabic renderings of the major Greek treatises—the original works appeared in print only around 1525—Achillini, Zerbi and Berengario worked hard along this line. They exploited a novel method of multiple citations of Greek and Arabs as well as various medieval scholars and, as such, they paved the way to Vesalius’ major reform of anatomical nomenclature.

These efforts to develop a coherent anatomical nomenclature should not be viewed as the result of a rejection of Arabic works, quite the contrary. Arabic/Islamic scholars have fruitfully filled the gap between Galen and the pre-Vesalian anatomists. Their formative influence has extended from the medieval period to the 17th century (Russell, 2010). We owe them a remarkable synthesis of the Greek and Hellenistic knowledge about the brain, the nerves and the senses, particularly the visual system, and the Latin versions of their texts became standard textbooks throughout Europe during centuries. They also provided the first schematic neurology-related diagrams that served as models for the medieval scholars (Choulant, 1920; Nutton, 2001; Russell, 2010). Their description of the functional organization of the motor and sensory systems by analogy with the mechanisms of hydraulic automata anticipated similar notions that appeared in the 17th century. Hence, despite the pre-Vesalian effort to eradicate Arab anatomical terminology, the notions developed by Arabic scholars in regard to the anatomical and functional organization of the nervous system remained at the heart of the Renaissance thinking, teaching and drawing.

Among the various pre-Vesalian anatomists of the second wave, Johannes Dryander of Marburg deserves a particular mention because he was first anatomist to devote an entire treatise to a single part of the human body, namely the head. His *Anatomia capitis humani* (Anatomy of the human head), first published at Marburg in 1536 (Dryander, 1537), is a dissection manual that comprises essentially the legends of 12 figures intended to describe the entire anatomy of the human head. The text is short and the illustrations rudimentary compared to those of Berengario, but the book nevertheless provides interesting dissection cues and, for the first time, illustrates the stages of a progressive dissection of the head and the brain (Russell, 2013). In the legend of figure VI, Dryander quotes a description of the buttocks (*anchae*) directly taken from Berengario’s *Isagogae* (see above). His figure XII, which can be seen as the take-home message of the entire work is rather disappointing. It offers a crude, schematic view of a human head “borrowed” from Hundt (see above) and in which emphasis is placed on the *rete mirabile* and the cerebral ventricles as the seat of higher brain functions. The other pre-Vesalian anatomists of the late contingent—de Laguna, Massa and Guenther—have complemented the overall study of human anatomy initiated by their predecessors, but none of them surpassed Berengario in regard to the description of the human brain.

## Concluding Remarks

It is hoped that this brief review will convince the reader that Jacopo Berengario da Carpi has played a catalyzing role in the transmutation of medieval morphological knowledge into a modern anatomical science based upon direct observation and experimental demonstration. This indefatigable physician, surgeon and anatomy teacher mustered enough courage to challenge Galen and to promote independent research in the field of human anatomy, including neuroscience. His remarkable treatise on skull fractures published 500 years ago has worth him the title of a pioneer in skull fracture surgery, whereas his Commentary on Mondino—a veritable treasure of rare information and new experiences—made him the founder of a new epoch in anatomy characterized by the supremacy of sensory over textual versions of the truth. Berengario was the first anatomist to recognize the value and significance of anatomical illustrations in clarifying the text and, in so doing, he paved the way to the anatomists who came after him, including the great Vesalius. Berengario embodies the transition from the uncritical repetition of old notions to reliance on empirical observation, a shift that led to the development of scientific anatomy.

## Author Contributions

The author confirms being the sole contributor of this work and has approved it for publication.

## Conflict of Interest Statement

The author declares that the research was conducted in the absence of any commercial or financial relationships that could be construed as a potential conflict of interest.
